# On Anesthetic Inhalations in Certain Painful Affections of the Chest

**Published:** 1853-09

**Authors:** 

**Affiliations:** Strasbourg


*On Anesthetic Inhalations in Certain Painful Affections of the Chest.* By Dr. Carriere, of Strasbourg. Dr. Carrière relates a case of convulsive cough, and one of angina pectoris, as examples of the benefits which are likely to result from the employment of anesthetic inhalations in certain painful affections of the chest.

*Case* 1. The subject of this case was a nervous girl, 19 years of age, without fever, organic pulmonary disorder, or any perceptible derangement of her health, except a violent spasmodic cough, chiefly at night. Simple means were applied, then morphia, balladonna, oil of bitter almonds, and other remedies were tried without success for fifteen days. She was next put partially under the influence of chloroform, twice in the course of one evening, wnen the cough ceased, not again to return.

*Case* 2. M. P—, æt. 64, previously in the enjoyment of good health, has suffered for some months from the symptoms of fully developed angina pectoris, his paroxysms being brought on by the slightest mental or physical exertion. The circulation is extremely feeble, and there is some œdema in the limbs, but the respiration is pretty free, and there is no particular mischief in the heart. During each paroxysm the lungs became considerably engorged, and after it, some viscid bloody mucus is expectorated. Blisters, synapisms, narcotic fumigations, belladonna, hemlock, digitalis, ether, quinia, and other stock remedies were tried for some time, without avail. Chloroform inhalations were then recommended, but the patient having an objection to them, ether was substituted, and with the same marked and immediate relief as in the former case. The instructions were for the patient himself to conduct the inhalation, and to begin as soon as he felt the threatenings of the paroxysm. The result was, that the threatenings became less and less frequent, and in two months disappeared altogether, and the patient was restored to a state of comparatively good health. It must be stated that the objections to chloroform were overcome in the course of the treatment, and this remedy having been substituted for the ether was preferred to it. —*Bulletin Gén. de Thérap.*, and *Nouvelle Encyclogr. des Science Medicale.*

